# Climate Change and Infectious Disease Risk in Western Europe: A Survey of Dutch Expert Opinion on Adaptation Responses and Actors

**DOI:** 10.3390/ijerph120809726

**Published:** 2015-08-18

**Authors:** Su-Mia Akin, Pim Martens, Maud M.T.E. Huynen

**Affiliations:** International Centre for Integrated assessment and Sustainable development (ICIS), Maastricht University, P.O. Box 616, 6200 MD Maastricht, The Netherlands; E-Mails: p.martens@maastrichtuniversity.nl (P.M.); m.huynen@maastrichtuniversity.nl (M.M.T.E.H.)

**Keywords:** climate change, public health, infectious diseases, climate change adaptation

## Abstract

There is growing evidence of climate change affecting infectious disease risk in Western Europe. The call for effective adaptation to this challenge becomes increasingly stronger. This paper presents the results of a survey exploring Dutch expert perspectives on adaptation responses to climate change impacts on infectious disease risk in Western Europe. Additionally, the survey explores the expert sample’s prioritization of mitigation and adaptation, and expert views on the willingness and capacity of relevant actors to respond to climate change. An integrated view on the causation of infectious disease risk is employed, including multiple (climatic and non-climatic) factors. The results show that the experts consider some adaptation responses as relatively more cost-effective, like fostering interagency and community partnerships, or beneficial to health, such as outbreak investigation and response. Expert opinions converge and diverge for different adaptation responses. Regarding the prioritization of mitigation and adaptation responses expert perspectives converge towards a 50/50 budgetary allocation. The experts consider the national government/health authority as the most capable actor to respond to climate change-induced infectious disease risk. Divergence and consensus among expert opinions can influence adaptation policy processes. Further research is necessary to uncover prevailing expert perspectives and their roots, and compare these.

## 1. Introduction

Warming in the climate system is unequivocal and human influence on the climate system is clear [1]. Climate change is impacting all areas of life, including human health. Also, within a European context, climate change will have impacts in nearly all sectors and all regions. In addition, the health effects of global warming are increasingly evident [2,3,4,5,6]. Among the anticipated health impacts, climate change-induced infectious disease risks pose a significant (future) health as well as social and economic burden on Europe [6,7]. The prospect of even further global warming [1] only increases concerns for these potential impacts. Climate change can bring about changes in the distribution and transmission of communicable diseases and, related to this, the number of disease cases by influencing e.g., the disease pathogen directly, the suitability of environments, or human behaviors leading to exposure [3,5,6,8]. Tick-borne encephalitis, leishmaniasis, and salmonellosis are, for example, relevant for Europe in this context of climate change impacts [3,6].

Climate change and the related health impacts pose a great challenge on our societies, and are therefore gaining increasing scientific and policy attention [9,10,11,12,13,14,15]. Subsequently, the call for adequate (policy) responses to address this challenge is becoming increasingly stronger, as illustrated by, for example, the European Commission White Paper “Adapting to climate change: Towards a European framework of action”; the “Declaration of the European Commission Fifth Ministerial Conference on Environment and Health”; and the recent European Commission Staff Working Document “Adaptation to climate change impacts on human, animal and plant health” [11,12,13,14]. Some of the observed and anticipated (health) effects from climate change in Europe (e.g., increased mortality due to high temperature extremes) have initiated the development and implementation of adaptation strategies [2].The (future) health risks and vulnerabilities arising from climate change, in interaction with other non-climatic drivers, can be reduced by adaptation responses. Current adaptation efforts should be improved and extended to reduce risk as far as possible. More understanding and research regarding responses and their effectiveness and health implications is necessary [5,12,13,16].

It is clear that a more effective (policy) response and an overall strengthening of health systems in Europe are needed to address climate change and the resulting health impacts [5,12,13]. A broad range of necessary aspects of such a response are discussed and put forward in related studies and literature, such as climate-sensitive surveillance and monitoring systems; outbreak investigation, response, and control; raising awareness and education of policy-makers, health professionals and public; information and communication systems for awareness raising; emergency preparedness and planning; research; access to health care and preventive measures; and cooperation and partnerships (see e.g., [2,3,4,5,6,17]).

In this study, conducted as a part of the ERA-ENVHEALTH ENHANCE research project, the perspectives of Dutch experts on such responses to climate change-induced infectious disease risk in Western Europe are explored. Moreover, experts’ views on the willingness and capacity of potentially relevant actors in this response effort as well as the priorities placed on mitigation and adaptation responses are examined (see [18]). The study has been conducted as a part of a participatory integrated assessment where the involvement of stakeholders (in this case, experts) is a way to address complexities and uncertainties that are common to environmental and specifically climate change problems [19,20]. The study, therefore, makes use of a stakeholder analysis for the identification and selection of the Dutch expert sample. For the analysis, an integrated view on the causality of infectious disease risk is taken on by including multiple climatic as well as non-climatic factors as drivers (see [21]). Based on this study, other results on Dutch expert opinion on climatic and non-climatic drivers of infectious disease risk in Western Europe have also been published (see [21]).

## 2. Methodology

This study was conducted to gain an insight into Dutch experts’ views on responses to climate change-induced infectious disease risk in Western Europe. A qualitative survey was used to uncover existing perspectives (and the possible diversity among them) in the Netherlands, by targeting a Dutch expert sample. In preparation for this, a stakeholder analysis was done to sample Dutch experts. The obtained data were analyzed descriptively. (For the methodology of this study, also see [21], where the survey results on climatic and non-climatic drivers of infectious disease risk are presented.)

### 2.1. Stakeholder Analysis and Sampling

A stakeholder analysis was done to identify experts for the selection of potential respondents for the survey used in this study [22,23]. A broad definition of stakeholders was assumed for this analysis: individuals and organizations that are likely to be affected by or can influence climate change and related health impacts; the latter e.g., through the implementation of adaptation measures [23,24,25,26]. The analysis was restricted to the Netherlands, being a priority research focus of the ERA-ENVHEALTH ENHANCE research project. The stakeholder analysis aimed to map out Dutch stakeholders in relation to the topic of climate change and infectious disease risk in Western Europe; to create a useful inventory for the potential participants for the participatory elements in the broader research setup of the project; and to facilitate the collection of expert knowledge and perspectives on climate change and health impacts. This approach to a stakeholder analysis can be characterized as descriptive and instrumental [23].

The stakeholder analysis yielded a stakeholder matrix storing detailed information for each stakeholder, such as the stakeholder’s expertise and professional background. Based on the stakeholder matrix, an expert sample of respondents for the survey could be selected. The guiding criterion for selecting a sample of experts from the stakeholder matrix was that the individuals possess expertise relevant for the survey’s topic and scope; *i.e*., expertise on climate change/environment, and health/infectious diseases. Information on the expertise of each stakeholder was documented in the stakeholder matrix and could therefore be retrieved for the purpose of sampling. Since a guiding criterion was used as a base for sampling selection, this sampling method can be called judgment sampling, a non-probabilistic sampling method [27,28]. From the stakeholder matrix a sample of 56 experts on climate change/environment and health/infectious diseases could be drawn. Based on the information collected about each stakeholder’s professional background, the expert sample included mostly scientists and policy advisors. The survey was conducted in 2012 and yielded a useful response of 29 (out of the 56 experts who were approached as respondents for the survey).

The survey included a section where respondents could indicate their professional background, using predefined categories with the possibility of adding additional categories in an open section. The data were used to divide the sample into groups. This was done to explore potential differences and similarities in opinions of experts with different professional backgrounds in the analysis. The sample groups were formed in the following manner: first, all respondents who indicated a professional background in policy were placed in one sample group labeled “Policy” and thereafter removed from the full list of respondents. After that, the respondents who indicated a professional background in science were included in another sample group labeled “Science”; finally, the remaining three respondents were added to the group “Policy” as the indicated professional backgrounds seemed to allow for such a compilation. This process resulted in two sample subgroups: “Policy,” consisting of 12 experts, and “Science,” consisting of 17 experts. As explained earlier, based on information collected in the stakeholder matrix on the professional backgrounds of the selected sample, the sample could be characterized as comprising scientists and policy advisors. The self-indicated professional backgrounds of the respondents leading to the formation of the sample subgroups confirms the initial characterization made of the composition of the selected sample. In this respect, the sample subgroups seem to be a sensible division of the aggregate sample.

### 2.2. Survey Design

This study aimed to have experts assess responses to climate change-induced infectious disease risk in Western Europe, and the capacity and willingness of potentially relevant actors in this context. A survey was used to accomplish this as this is a suitable and efficient method for a qualitative assessment based on expert opinions and perceptions [27]. The survey design included closed questions, mostly in a Likert-scale format, and optional open questions. Each closed question provided a possibility to leave it open (“no opinion/do not know”) and to add more items that could then be assessed as well. All definitions could be viewed by the respondents at all times during the survey process. The survey was administered online and generated descriptive information, nominal, ordinal, and some ratio/interval data.

Using a survey method involves several common limitations, which were taken into account during the design and implementation phases of this study. For instance, the risk of misunderstanding of wording [27] was reduced by using vocabulary familiar to experts and making definitions available at all times during the survey process. Research design measures, such as using an expert sample and offering a “no opinion/don’t know” option, could help to safeguard the quality of responses, which can be another potential risk of survey methods.

### 2.3. Survey Content

The survey content was structured in the following manner: the assessment of

(a) Adaptation responses to climate change-induced infectious disease risk in Western Europe;
(b) The priorities under budgetary constraints for mitigation or adaptation responses to climate change-induced infectious disease risk; and
(c) The willingness and the capacity of potentially relevant actors to respond to climate change-induced infectious disease risk.

In part (a) of the survey, experts assessed adaptation responses to climate change-induced infectious disease risk in Western Europe, using pre-defined policy assessment criteria. Based on descriptive research and background literature, the possible and most relevant adaptation responses to climate change impacts on infectious disease risk could be selected [3,17]. The responses assessed in the survey are also acknowledged (in the same or a similar form) in relevant European policy documents [12,13,14,29]. In Table 1 an overview of the responses used in the survey is given.

**Table 1:** Descriptions/definitions of the responses to climate change-induced infectious disease risk used in the survey.

Response	Description
Monitoring	Includes both indicator-based surveillance and event-based epidemic intelligence. Indicator-based surveillance refers to data collection, (trend) analysis, and interpretation of data. Indicator-based surveillance is conducted based on data from infectious disease notifications at the European Union level, pharmacy-based monitoring, sentinel surveillance, vector distribution monitoring, real-time surveillance, monitoring cause-specific deaths from infectious diseases, and syndrome surveillance. Event-based epidemic intelligence refers to the early identification of climatic change-induced infectious disease threats, through screening of news media, scientific reports, clinical case reports, and interdisciplinary reporting (such as from related industrial sectors) [3].
Outbreak investigation and response	The diagnosis and investigation of new/emerging infectious diseases, as well as responding effectively to (potential) outbreaks involving the cooperation of multiple sectors. For effective response adequate supplies (such as medication and vaccinations) and distributive infrastructures are required and thus need to be included in this sort of policy option [3].
Dissemination of information, and health education	The communication of information, health education, and health promotion; all relating specifically to human (risk) behavior in the context of work, recreational activities, food handling, but also personal preventive measures for vector control and minimization of exposure [3].
Foster interagency and community partnerships	Fostering interagency and community partnerships, thus establishing partnerships between stakeholders across different sectors, disciplines, and levels (e.g., local community, municipality, national) [3].
Enforce laws and regulations	Enforcing laws and regulations regarding reporting climate change-induced health events, setting up and enforcing conventions relating to e.g., water resources and sanitation, and regulation to e.g., minimize harmful exposures [3].
Access to health care, and prevention	Providing access to health services, prevention of outbreak propagation through treatment of infected patients, and prevention (such as vaccination, prophylaxis, travel medicine) [3].
Strengthen capacity of public health workforce and implement emergency preparedness training	Strengthening of the capacity (in terms of quality and quantity) of public workforce for instance through training focused on diagnosis of specific infectious diseases. In addition, this policy option includes the implementation of emergency preparedness training [3].
Research	Fostering research focused on the relationship between infectious diseases and climate change, risk mapping of climate sensitive vectors and pathogens, development of diagnostic tests and vaccines, burden-of-disease studies, and climate as well as policy scenarios, amongst other related research activities [3].
Environmental management/modification	The practice of using environmental management to reduce the capacity of local habitats to maintain pathogens and/or populations of disease vectors (e.g., reducing reservoir host abundance, removing larval breeding sites), thus resulting in a decrease of spread of a disease to humans/animals [17].
Direct control methods	The use of chemical or biological control methods in order to reduce pathogen or vector abundance. Chemical control methods consist of, for example, the use of insecticides or water treatment chemicals. Biological control methods consist of the utilization of biological toxins and natural enemies include e.g., bacteria and larvivorous fish [17].

In order for experts to assess the selected responses, eight policy assessment criteria were defined, partly based on a previous relevant study by the World Health Organization [30]. These criteria are given in Table 2. In the survey, therefore, each response was assessed using all eight assessment criteria.

**Table 2:** Descriptions/definitions of the assessment criteria used in the survey.

Assessment Criterion	Description
Potential health gain	The “potential magnitude of the health gain” of the policy option [30] (p.23).
Uncertainty of potential health gain	The extent to which the potential health impact (*i.e.,* gain) of the policy option is uncertain. Uncertainty refers to the predictability of the health outcomes that can result from the policy option [30].
Monetary costs	Direct and indirect monetary costs of the policy option [30].
Non-monetary costs	The potential (non-monetary) imposition on the health system and society at large that this policy option can create
Positive spill-over effects	The potential that this policy option can bring about positive side-effects for other policy areas, sectors, and/or for society
Flexibility	The degree of ease for modification of the policy option should this become necessary in the future [30].
Urgency of implementation	The rapidity at which the policy option needs to be implemented for it to still have an effect, and if it is not to run into any implementation barriers [30].
Regret if climate change does not turn out as expected	The extent that the policy option will still be effective in terms of health gain if climate change trends turn out differently than expected [30].

In part (b) of the survey the priorities placed by the expert sample on mitigation and adaptation measures as part of an overall response strategy were assessed. Responses to climate change impacts can be broadly grouped into two categories, adaptation or mitigation measures. For the purpose of the survey the following definitions were assumed: Mitigation responses are measures to reduce greenhouse gas emissions and enhance sinks in order to mitigate anthropogenic climate change; and adaptation responses are initiatives and measures to reduce climate change-induced infectious disease risk [31].

Finally, in part (c), the survey aimed to obtain expert views on the willingness and the capacity of potentially relevant actors to respond to climate change-induced infectious disease risk. For this, the following actor categories were formulated: national government/health authority; local health authority; policy-advice, policy-maker; NGO, advocacy, funders, charity; science; health care provider/health practitioner; veterinary profession; pharmaceutical industry; farmer union; food supply sector; environmental management, conservation; trade and tourism sector; and other business/private sector actors (ENHANCE Research Project, see [32]).

### 2.4. Survey Analysis

The resulting survey data were analyzed using descriptive statistics. The statistics performed are appropriate for the ordinal data obtained from Likert-scale questions. Thus, for the analysis of central tendency and variability median, mode, range, and inter-quartile range were used (see e.g., [27]). For the ratio data obtained from part (c) of the survey, the median and standard deviation were used for the analysis of central tendency and variability. 

Moreover, the smaller sample size and non-probabilistic sampling method were taken into account for the analysis. Uncompleted surveys were excluded from the analysis. For the analysis, the “no opinion/do not know” responses were assumed to be distributed proportionally amongst the respondents, and therefore were excluded in order to circumvent an inflation of the actual resulting responses [27].

In order to explore the experts’ opinions according to the experts’ self-indicated professional background, all descriptive analyses were done for the aggregate sample as well as for the two sample groups “Policy” and “Science” described earlier.

## 3. Results

In this section the results of the survey are presented in the form of descriptive statistics, following the structure of the survey, parts (a)–(c), as explained earlier in the methodology.

### 3.1. Expert Assessment of the Responses to Climate Change-Induced Infectious Disease Risk

**Figure 1:**
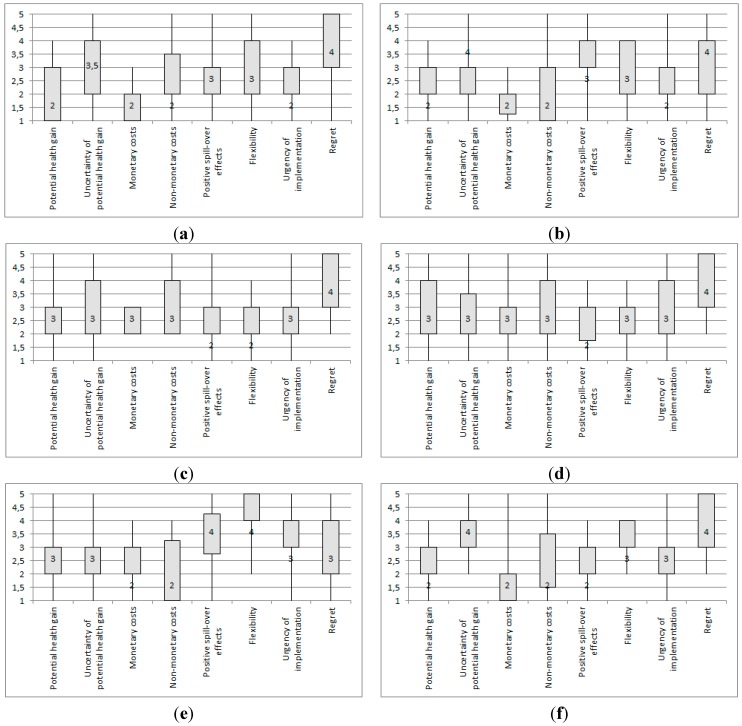

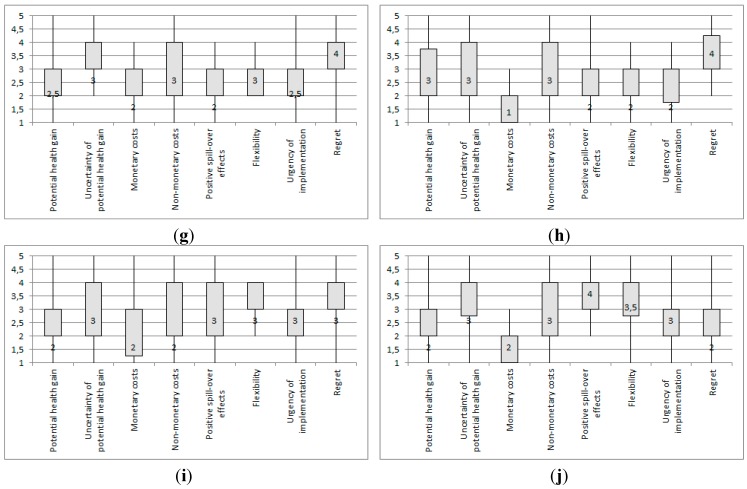
Box plots of the aggregate expert sample assessment of the responses to climate change-induced infectious disease risk in Western Europe, using the eight assessment criteria:(**a**)monitoring; (**b**) outbreak investigation and response; (**c**) dissemination of information, and health education; (**d**) fostering interagency and community partnerships; (**e**) enforcing laws and regulations; (**f**) access to health care, and prevention; (**g**) strengthening the capacity of public health workforce and implement emergency preparedness training; (**h**) research; (**i**) environmental management; and (**j**) direct control methods.Notes: Medians are given as numbers in the box plots; range = maximum value − minimum value; interquartile range (IQR) = 3rd quartile − 1st quartile = length of box. Interpretation of median values for each assessment criterion: Potential health gain: 1 = very high *(high, moderate, low)*; 5 = very low; uncertainty of potential health gain: 1 = very high uncertainty *(high, moderate, little)*; 5 = virtually certain; monetary costs: 1 = high costs *(moderate costs, low costs, no or negligible costs)*;5= net benefits; non-monetary costs: 1= high costs *(moderate costs, low costs, no or negligible costs)*;5 = net benefits; positive spill-over effects: 1 = very high *(high, moderate, low)*; 5 = very low to none; flexibility: 1 = very high flexibility *(high, moderate, low)*; 5 = no flexibility; urgency of implementation: 1= very high urgency *(high, moderate, little)*; 5 = no urgency; regret if climate change does not turn out as expected: 1 = very high potential regret if climate change does not turn out as expected *(high, moderate, little)*; 5 = no regret if climate change does not turn out as expected.

**Figure 2:**
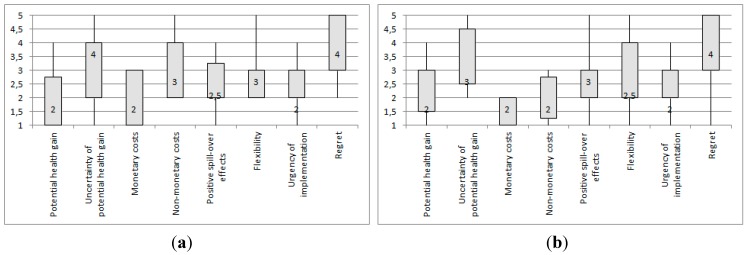

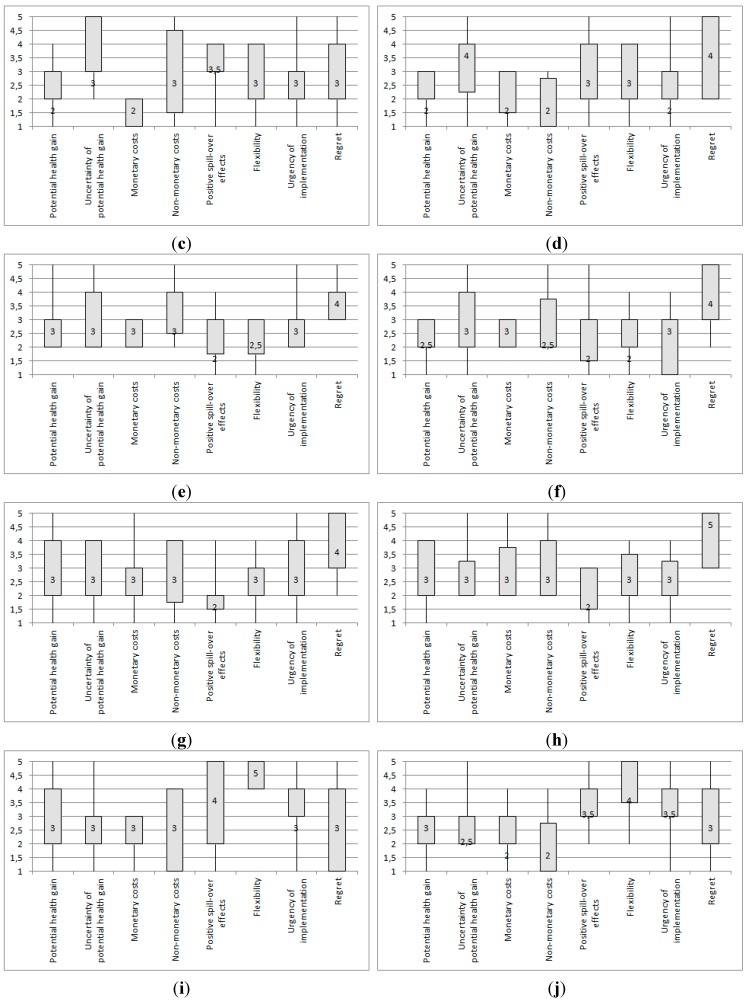

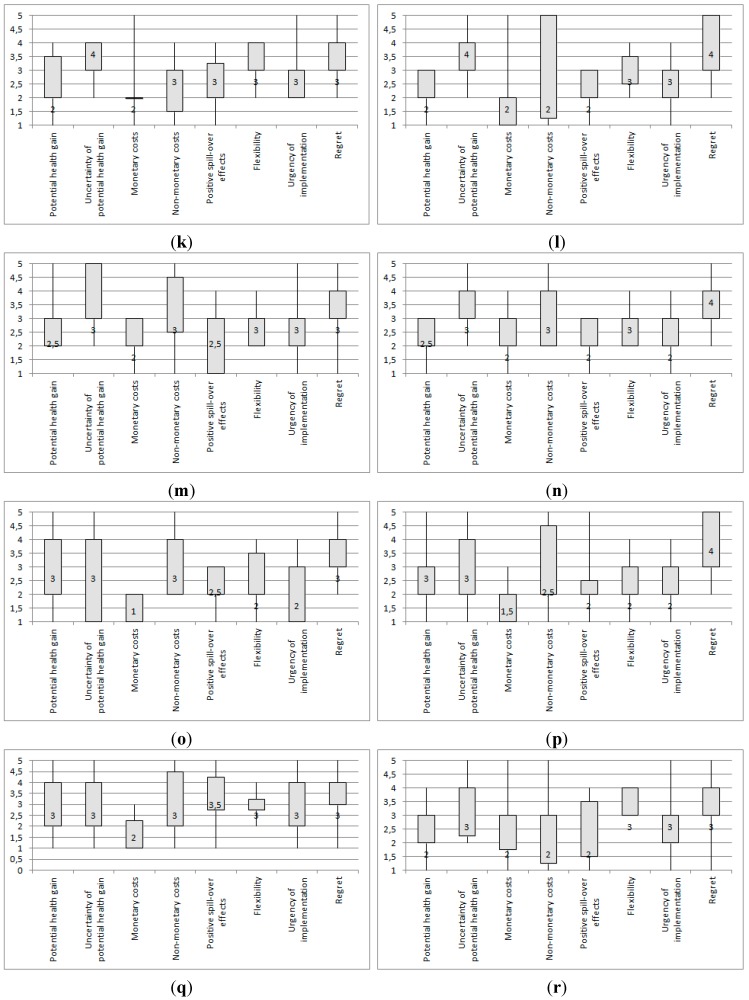

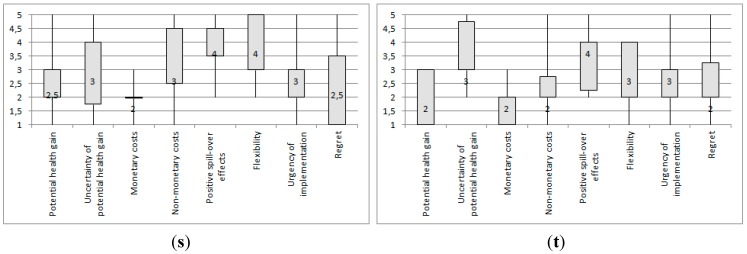
Box plots of the assessment of the two sample groups “Policy” and “Science” of the responses to climate change-induced infectious disease risk in Western Europe, using the eight assessment criteria:(**a**) Monitoring, sample group “Policy”; (**b**) monitoring, sample group “Science”; (**c**) outbreak investigation and response, sample group “Policy”; (**d**)outbreak investigation and response, sample group “Science”; (**e**) dissemination of information, and health education, sample group “Policy”; (**f**) dissemination of information, and health education, sample group “Science”; (**g**) fostering interagency and community partnerships, sample group “Policy”; (**h**) fostering interagency and community partnerships, sample group “Science”; (**i**) enforcing laws and regulations, sample group “Policy”; (**j**) enforcing laws and regulations, sample group “Science”; (**k**) access to health care, and prevention, sample group “Policy”; (**l**) access to health care, and prevention, sample group “Science”; (**m**) strengthening the capacity of public health workforce and implement emergency preparedness training, sample group “Policy”; (**n**) strengthening the capacity of public health workforce and implement emergency preparedness training, sample group “Science”; (**o**) research, sample group “Policy”; (**p**) research, sample group “Science”; (**q**)environmental management, sample group “Policy”; (**r**) environmental management, sample group “Science”; (**s**) direct control methods, sample group “Policy”; and (**t**) direct control methods, sample group “Science.”Notes: Medians are given as numbers in the box plots; range = maximum value − minimum value; interquartile range (IQR) = 3rd quartile − 1st quartile = length of box. Interpretation of median values for each assessment criterion: Potential health gain: 1 = very high *(high, moderate, low)*; 5 = very low; uncertainty of potential health gain: 1 = very high uncertainty *(high, moderate, little)*; 5 = virtually certain; monetary costs: 1 = high costs *(moderate costs, low costs, no or negligible costs)*;5= net benefits; non-monetary costs: 1= high costs *(moderate costs, low costs, no or negligible costs)*;5 = net benefits; positive spill-over effects: 1 = very high *(high, moderate, low)*; 5 = very low to none; flexibility: 1 = very high flexibility *(high, moderate, low)*; 5 = no flexibility; urgency of implementation: 1= very high urgency *(high, moderate, little)*; 5 = no urgency; regret if climate change does not turn out as expected: 1 = very high potential regret if climate change does not turn out as expected *(high, moderate, little)*; 5 = no regret if climate change does not turn out as expected.

Through analysis of the survey data, the expert sample’s assessment of the adaptation responses to climate change-induced infectious disease risk in Western Europe(defined in Table 1), making use of the predefined eight assessment criteria (defined in Table 2), could be obtained. The results of the aggregate sample analysis are presented first in Figure 1(a)–(j), and thereafter of the two sample groups “Policy” and “Science” in Figure 2(a)–(t).

Figure 1(a)–(j) shows the results of the aggregate expert sample’s assessment of the adaptation responses using the eight criteria.

Several notable results can be gathered from the box plots in Figure 1 (a)–(j). The following responses are rated by the aggregate sample as having high potential health gains: “monitoring,” “outbreak investigation and response,” “access to health care and prevention,” “environmental management,” and finally “direct control methods”. However, only for “outbreak investigation and response” and “access to health care and prevention”, there is little uncertainty of this potential health gain according to the expert sample. For “monitoring,” “environmental management,” and “direct control methods,” the IQRs for the uncertainty of the potential health gain are relatively greater, indicating that the responses of the aggregate sample vary.

A number of responses are believed by the experts to involve low costs. “Dissemination of information and health education” and “foster interagency and community partnerships” have low monetary and non-monetary costs according to the experts. For “strengthen capacity of public health workforce and implement emergency preparedness training” and “direct control methods” low non-monetary costs are found. The experts view “research” as having high monetary costs, but low non-monetary costs. The IQRs are relatively high in all instances for non-monetary costs, indicating that there is quite some variance in the responses of the experts on this criterion.

For “dissemination of information and health education”, “foster interagency and community partnerships”, “access to health care and prevention”, “strengthen capacity of public health workforce and implement emergency preparedness training”, and “research,” the positive spill-over effects are seen by the expert sample as high. Positive spill-over effects are rated as low for “direct control methods” and “enforce laws and regulations”. Generally, the IQRs for the results on positive spill-over effects are relatively lower, indicating less variability amongst the responses of the expert sample on this criterion. One exception is “environmental management”, where the IQR and thus the variation in the answers for positive spill-over effects are relatively greater.

With regards to flexibility, the results show that “dissemination of information and health education” and “research” are seen as highly flexible, but “enforce laws and regulations” has low flexibility. “Monitoring”, “outbreak investigation and response,” and “research” are rated as highly urgent by the expert sample. Finally, when taking a look at the results for regret if climate change does not turn out as expected, the expert sample assesses all responses to have little regret, except for “environmental management” and “direct control methods”, where the latter is even seen as having high regret. It should be noted that the IQRs for regret are relatively greater for the majority of the responses, which indicates that the aggregate sample’s answers vary for regret.

The same analysis was done for the two sample groups, “Policy” and “Science”. Figure 2 (a)–(t) shows the assessment of adaptation responses to climate change-induced infectious disease risk in Western Europe using the eight assessment criteria, according to the sample groups “Policy” and “Science”.

The results in the box plots in Figure 2 (a)–(t) show that the sample groups “Policy” and “Science” mostly agree when it comes to the following assessment criteria: potential health gain, uncertainty of potential health gain, monetary costs, flexibility, and urgency of implementation. Most differences between the two sample groups in terms of assessment criteria can be seen for non-monetary costs, positive spill-over effects, and regret if climate change turns out differently than expected. A relatively greater difference between the assessments of the sample groups can be seen for the responses “outbreak investigation and response” and “enforce laws and regulations”. Much agreement between the assessments of the two groups can be found for “dissemination of information, and health education”, “foster interagency and community partnerships”, “access to health care, and prevention”, “strengthen capacity of public health workforce and implement emergency preparedness training”, and “environmental management”.

The group “Policy” shows greater IQRs for potential health gain, uncertainty of health gain, and non-monetary costs, which means that greater variation in the responses for these criteria is found within this sample group. For the group “Science”, greater IQRs and thus more variation in responses could be found for the criteria uncertainty of health gain, non-monetary costs, and regret if climate change turns out differently than expected. The group “Policy” shows a greater amount of relatively higher IQR values in the assessment of the responses “monitoring,” “foster interagency and community partnerships,” “research”, and “environmental management.” The group “Science” shows a greater amount of relatively higher IQR values in the assessment of the responses “monitoring,” “outbreak investigation and response”, “dissemination of information, and health education,” and “foster interagency and community partnerships”.

### 3.2. Expert Prioritization of Mitigation and Adaptation Response Strategies

The respondents were asked to divide (in percentages adding up to a total of 100%) a fictional limited budget between mitigation and adaptation responses to address climate change-induced infectious disease risk in the most optimal manner. In Table 3 the results of the analyses of the responses of the aggregate sample as well as the sample groups “Policy” and “Science” are shown.

**Table 3:** Assessment of the percentage committed to mitigation and adaptation response strategies under budgetary constraints, by the aggregate sample and the two sample groups “Policy” and “Science”.

	Aggregate Sample		Sample Group “Policy”		Sample Group “Science”	
Response Strategy	Mean (in %)	Standard Deviation	Mean (in %)	Standard Deviation	Mean (in %)	Standard Deviation
Mitigation	50.52	27.37	47.92	24.63	52.35	29.75
Adaptation	49.48	27.37	52.08	24.63	47.65	29.75

Notes: IQR = Interquartile range = 3rd quartile − 1st quartile. In part (c) of the survey the respondents could give their answers in percentages, resulting in ratio data. Therefore, the mean and standard deviation are used for the analysis of central tendency and variation, which are appropriate measures for ration/interval data (see e.g. [27]) (as described in the methodology).

The mean percentage assigned by the aggregate sample to mitigation is 50.52% and to adaptation is 49.48%. Based on these results, it would seem that there is no clear priority placed on either mitigation or adaptation measures, so a (policy) response strategy including an equally proportioned mix of the two is shown in the results. When taking a closer look at the data resulting from the aggregate sample’s responses, it can be seen that indeed a large part of the sample opted for a 50/50 budgetary division between mitigation and adaptation options. However, divisions of 70%/30% and 80%/20% (for mitigation and adaptation, respectively) have been opted for relatively more frequently by the expert sample.

Additionally, the mean results for mitigation and adaptation have been assessed for the groups “Policy” and “Science”. Here a slight difference in emphasis between mitigation and adaptation results between the two sample groups can be found. The group “Policy” allocates a higher mean percentage of the budget to adaptation, whereas the group “Science” devotes a slightly higher mean percentage to mitigation (47.92% or52.35% for mitigation, and 52.08% or47.65% for adaptation, respectively). The standard deviations reveal that the sample group “Science” shows greater variance in their responses in comparison to the group “Policy”. Again, when taking a closer look at the dataset resulting from this question, the group “Policy” showed the same frequency of responses for the budgetary divisions of 30%/70%, 50%/50%, and 70%/30% (for mitigation and adaptation, respectively). For the sample group “Science”, a large portion of the respondents chose 50%/50%, but 80%/20% (mitigation/adaptation) also received considerable support.

### 3.3. Expert Assessment of Relevant Actors’ Willingness and Capacity to Respond

Next, the survey had the expert sample assess the willingness and the capacity to respond to climate change-induced infectious disease risk in Western Europe of potentially relevant actors (earlier defined as: national government/health authority; local health authority; policy-advice, policy-maker; NGO, advocacy, funders, charity; science; health care provider/health practitioner; veterinary profession; pharmaceutical industry; farmer union; food supply sector; environmental management, conservation; trade and tourism sector; other business/private sector actors). The results of the survey are first presented for the analysis of the actors’ willingness to respond by the aggregate sample and the two sample groups “Policy” and “Science” in Table 4 and thereafter the results will be shown for the capacity to respond of the actors in Table 5.

**Table 4:** Assessment of the willingness to respond of the actors, by the aggregate sample and the two sample groups “Policy” and “Science”.

Actors	Assessment of Willingness to Respond					
	Aggregate Sample		Sample Group “Policy”		Sample Group “Science”	
	Median	IQR	Median	IQR	Median	IQR
National government/health authority	3	2	3	1	3	2
Local health authority	3	2	3	2	2	2
Policy-advice, policy-maker	3	1	3	0	3	1
NGO, advocacy, funders, charity	2	1	2	1	2	1
Science	2	2	2	1	2	1.75
Health care provider/health practitioner	3	2	3	2	3	1.25
Veterinary profession	3	2	3	2	3	1.75
Pharmaceutical industry	3	1	2	2	4	2
Farmer union	4	1	4	1.5	4	2
Food supply sector	4	1	3	1.75	4	1.5
Environmental management, conservation	2	1	2	0	3	1
Trade and tourism sector	3	2	2	2	4	1
Other business/private sector actors	4	1	3	2.5	4	0

Notes: IQR = Interquartile range = 3rd quartile − 1st quartile. Interpretation of median values for willingness to respond: 1 = very high willingness to respond *(high, moderate, low)*; 5 = no willingness to respond.

**Table 5:** Assessment of the capacity to respond of the actors, by the aggregate sample and the two sample groups “Policy” and “Science”.

Actors	Assessment of Capacity to Respond					
	Aggregate Sample		Sample Group “Policy”		Sample Group “Science”	
	Median	IQR	Median	IQR	Median	IQR
National government/health authority	2	1	3	1	2	1
Local health authority	3	2	3	2	3	1.75
Policyadvice, policy-maker	3	1	3	2	3	1
NGO, advocacy, funders, charity	3	1.25	4	2	3	1
Science	3	1	3	1	3	1
Health care provider/health practitioner	3	2	2	1	3	1.75
Veterinary profession	3	2	2	1.5	3	1.75
Pharmaceutical industry	3	2	3	1.25	3	2
Farmer union	3	2	3	1	3	2
Food supply sector	3	2	3	2	3.5	1.75
Environmental management, conservation	3	2	3	2	3	1
Trade and tourism sector	3	2	2	1	3.5	1
Other business/private sector actors	3	1.5	4	2.25	3	1.5

Notes: IQR = Interquartile range = 3rd quartile − 1st quartile. Interpretation of median values for capacity to respond: 1 = very high capacity to respond *(high, moderate, low)*; 5 = no capacity to respond.

Table 4 shows the results of the aggregate expert sample’s assessment and the assessments of the two sample groups “Policy” and “Science” of the willingness to respond of the actors.

From the results in Table 4 it can be gathered that the aggregate sample rates the following actors as having a high willingness to respond: “NGO, advocacy, funders, charity”, “science”, “environmental management, conservation”, and the “trade and tourism sector”. The IQRs are also relatively smaller for the assessment of willingness of “NGO, advocacy, funders, charity” and “environmental management, conservation”, indicating that the responses of the aggregate sample for these two types of actors have relatively little variation. The aggregate sample’s responses yield low willingness to respond for the actors “farmer union”, “food supply sector”, and “other business/private sector actors”, all with relatively lower IQRs and thus relatively little variation in the responses of the aggregate sample.

The sample group “Policy” associates a high willingness to respond with the actors “NGO, advocacy, funders, charity”, “science”, the “pharmaceutical industry”, “environmental management, conservation”, and the “trade and tourism sector”. In the cases of “NGO, advocacy, funders, charity”, “science”, and “environmental management, conservation”, lower IQRs are also found, thus the sample group has relatively little variance in its responses. The group “Policy” indicates that “farmer union” is expected to have a low willingness to respond. This response also has a lower IQR.

The actors “local health authority”, “NGO, advocacy, funders, charity”, and “science” are rated as having high willingness to respond by the sample group “Science”. For “NGO, advocacy, funders, charity”, a lower IQR could be found. It can been seen that the two sample groups agree with regards to “NGO, advocacy, funders, charity” and “science” being actors with high willingness to respond, but differ in their assessment with regards to “local health authority”, the “pharmaceutical industry”, “environmental management, conservation”, and the “trade and tourism sector”. The sample group “Science” rates a greater number of actors as having low willingness to respond: the “pharmaceutical industry”, “farmer union”, the “food supply sector”, the “trade and tourism sector”, and “other business/private sector actors”, of which the latter two also receive a lower IQR. In comparison to the group “Policy”, the group “Science” sees more actors as having low willingness to respond; the two groups agree on the low willingness to respond of the actor “farmer union”.

In Table 5 the results of the assessments of the aggregate sample and the sample groups “Policy” and “Science” of the capacity to respond of the actors can be found.

From the results in Table 5 it becomes clear that the aggregate sample rates the “national government/health authority” as an actor with high capacity to respond. For this also a lower IQR can be found, which indicates that the aggregate sample’s responses vary relatively little.

The sample group “Policy” associates a high capacity to respond with the following actors: “health care provider/health practitioner”, the “veterinary profession”, and the “trade and tourism sector”. All these responses are also accompanied by a relatively lower IQR. The group “Policy” views the actors “NGO, advocacy, funders, and charity”, and “other business/private sector actors” as having a low capacity to respond. However, the IQR for the assessment of the capacity of “other business/private sector actors” is slightly greater, thus showing that the sample group “Policy” has some variation in responses for this item.

The group “Science” rates the “national government/health authority” as an actor with a high capacity to respond. Also, a lower IQR could be found here, so the sample group “Science” has little variation in their response. The two sample groups show no agreement on which actors have high or low capacity to respond (but are in agreement for some actors that are viewed as having a moderate capacity to respond).

## 4. Discussion and Conclusions

The survey presented in this paper aimed to shed light on the perspectives of Dutch experts on responses to climate change-induced infectious disease risk in Western Europe, the willingness and capacity of potentially relevant actors in this response effort, and finally the prioritization of mitigation and adaptation responses. The results of the survey and the analyses of the responses of the aggregate sample, as well as the two sample groups “Policy” and “Science,” reveal some information about the perspectives the experts hold. In summary, several key results have surfaced from the analyses.

The results show that the expert sample views the assessed responses differently in terms of the given criteria. There are two responses that are perceived by the experts as having both a high potential health gain and little uncertainty, “outbreak investigation and response” and “access to health care and prevention”. Additionally, “access to health care and prevention” is seen by the aggregate sample as having high positive spill-over effects. According to the expert sample, “dissemination of information and health education” and “foster interagency and community partnerships” have low monetary and non-monetary costs. It should be noted that the IQRs for non-monetary costs are relatively higher in all analyses, and therefore there may be less consensus among the experts in the aggregate sample as to the non-monetary costs for each of the responses assessed. Next to the low costs, “dissemination of information and health education” is also viewed as a highly flexible response with high positive spill-over effects. Also for “foster interagency and community partnerships,” high positive spill-over effects result from the survey. “Research” is viewed as a high monetary cost response but at the same time one with high positive spill-over effects, flexibility, and urgency. However, the majority of the results for “research” come with relatively greater IQR values, indicating that the aggregate sample shows variations in their responses for “research”. Overall, relatively larger IQRs for the criteria uncertainty of potential health gain, non-monetary costs, and regret if climate change does not turn out as expected can be found. This can indicate that the aggregate expert sample has fewer shared opinions on the issues of uncertainty, non-monetary cost, and regret. The assessments of some responses show overall relatively more variation than others: “foster interagency and community partnerships,” “research,” and “environmental management.” Thus the experts seem to have more diverging perspectives on these responses. The experts show the greatest consensus on their assessment of the responses “access to health care, and prevention” and “strengthen capacity of public health workforce and implement emergency preparedness” (based on the IQRs). The larger variations regarding several criteria and responses may also possibly relate to the idea that more understanding of response measures is needed as argued by some (see [5], among others).

When analyzing the results of the two sample groups “Policy” and “Science” it can be noted that the closest agreement between the assessments of the two groups can be found for “dissemination of information, and health education”, “foster interagency and community partnerships”, “access to health care, and prevention”, “strengthen capacity of public health workforce and implement emergency preparedness training”, and “environmental management”. The two groups seem to have differences in their assessments of the responses “outbreak investigation and response” and “enforce laws and regulations”. The perspectives within the group “Policy”, based on the resulting IQRs, seem to diverge most for the responses “monitoring”, “foster interagency and community partnerships”, “research”, and “environmental management”. For the group “Science” this is the case for “monitoring”, “outbreak investigation and response”, “dissemination of information, and health education”, and “foster interagency and community partnerships”. Within the “Policy” group, the greatest consensus could be found for the responses “access to health care, and prevention” and “strengthen capacity of public health workforce and implement emergency preparedness training.” Within the “Science” group, the greatest consensus could be found for the responses “enforce laws and regulations,” “access to health care, and prevention”, “strengthen capacity of public health workforce and implement emergency preparedness training,” and “research”.

Based on these analyses, one cannot deem one response to be better or worse than another. Important to note are those cases where the experts seem to show great divergence or consensus (based on the IQRs), which can indicate to what extent the results show a more or less shared perspective on the assessed response in question. In practice, adaptation responses are highly context-specific, and should ideally take into account both relevant climatic and non-climatic developments [33]. The priorities characterizing a decision-making context will influence which responses will be most fitting for the situation at hand. The criteria used in the survey can be translated to such possible decision-making priorities. For instance, when low monetary costs and high flexibility are important criteria for the decision on a response, then the “dissemination of information and health education” would, in the views of the aggregate expert sample, be a relatively more effective response in such a decision-making context. In this case, the answers for monetary costs and flexibility have relatively smaller ranges/IQRs, thus indicating greater agreement amongst the experts in the aggregate sample. When these guiding priorities change for another situation, this may lead to other choices in terms of response. The assessment criteria used in the survey cover a broad range of possible priorities for responses. In practice, some criteria for making decisions on adaptation measures might take precedence over others. For example, monetary costs are recognized as a key barrier to adaptation [13,33]. Due to this, more weight may be given to the criterion of low monetary costs, affecting the choice for an adaptation response strategy. In related studies it is argued that cost-effectiveness as well as no or little regret of adaptation response options can be priority characteristics in a decision-making context on adaptation [33,34].

Part (b) of the survey focused on prioritizing mitigation and adaptation responses under a fixed budget. The analysis of the results of the aggregate sample and the two sample groups “Policy” and “Science” all seem to indicate that a 50/50 budgetary allocation between mitigation and adaptation responses is the most optimal approach to tackle climate change-induced infectious disease risk in Western Europe. For the sample group “Policy,” however, the budgetary allocations of 30%/70% and 70%/30% (between mitigation and adaptation, respectively) were also opted for with the same frequency as a 50/50 division. Overall, relatively more variation in the responses of the “Science” sample group could be found. A response strategy including both mitigation and adaptation measures is acknowledged to be crucial for tackling (future) climate change consequences optimally [2].

The results on the assessment of the willingness and the capacity to respond, displayed in Table 4 and Table 5, show that the aggregate expert sample views the “national government/health authority” as the most capable actor to respond to climate change-induced infectious disease risk, and the lower IQR indicates that the experts show high agreement on this. The aggregate expert sample sees the actors “NGO, advocacy, funders, charity”, “environmental management, conservation”, and the “trade and tourism sector” as having a high willingness to respond. The results on “NGO, advocacy, funders, charity” and “environmental management, conservation” are also accompanied by lower IQRs, indicating that there is greater consensus among the expert opinions on the willingness of these actors. Lower willingness to respond is associated by the aggregate sample with the actors “farmer union”, the “food supply sector”, and “other business/private sector actors”, also with lower IQRs. The sample groups “Policy” and “Science” show no agreement with regards to which actors have a high or low willingness to respond. For high and low capacity to respond, the two sample groups show some agreement but also some differences.

The survey results should be seen as an indication of the perspectives of Dutch experts on the responses to climate change-induced infectious disease risk in Western Europe, and the willingness and capacity of the actors in such a response effort, based on the Dutch expert sample that participated in this study. The approach to conduct a participatory integrated assessment for the ERA-ENVHEALTH ENHANCE research project, of which this survey was a key research component, allowed for the involvement of stakeholders (the expert sample) to address the complexities and uncertainties inherent to the topic of climate change and infectious disease risk. The exploration of expert perspectives can provide insights in the complexity of climate change (and other environmental topics), as well as the related policy field [19,20,21]. The involvement of stakeholders, specifically for the assessment of adaptation responses, is argued as a necessary approach, also for future research efforts in this area [33,34]. Currently there is a lack of guidelines for the assessment of policy options for climate adaptation decision-making [30]. The use of expert perspectives to gain more insight into the responses assessed according to a broad range of criteria can help to address this need. In addition to the set of criteria used in this study, other criteria for the assessment of adaptation responses can be formulated.

The survey only relied on the answers provided by the Dutch expert sample, which should be kept in mind for the interpretation of the results of this study. Similar studies involving experts from different countries could be conducted to compare and contrast potentially prevailing perspectives on responses to climate change-induced infectious disease risk in Western Europe and the willingness and capacity of relevant actors in such a response [21]. Moreover, the composition of the aggregate sample, including scientists and policy advisors, could have influenced the results, and further studies incorporating various stakeholders could help to establish whether the responses are influenced by the composition of the sample. It should also be noted that this study focused on Western Europe as a geographical area. The disease burden and the role of climate change and infectious disease risk in this varies greatly across regions and countries and is often tied in with income levels and other non-climatic conditions. Therefore, the disease burden in Western Europe from climatic drivers for infectious disease risk is comparatively limited [35]. The results of the study should be interpreted with this background in mind.

The results of the analyses of the two sample groups “Science” and “Policy” can indicate that the two professional environments possibly have differing opinions when it comes to certain responses, assessment criteria, and the willingness and capacity of the actors. These differences could be related to the diversity in underlying values held in the two professional environments [21,36]. To establish prevailing perspectives on responses to climate change and infectious disease risk and make comparisons across professional backgrounds, more research involving experts needs to be done. In addition, the potential role of other factors contributing to differences in perspectives should be considered as well, such as possible information asymmetries between scientific and policy communities [21]. Differences in perspectives within and between sample groups can indicate a lack of consensus with regards to several responses and the assessed actors, which can have implications for policy processes for climate change adaptation. Increasing our awareness of the diversity in values and related perspectives as well as understanding the underlying (other) drivers of the perspectives can support policy processes for the adaptation to climate change-induced infectious disease risk. Responses that accommodate the prevailing perspectives of a broad range of stakeholders could benefit from greater support [21]. It would therefore be interesting to perform the same survey targeting samples of other relevant stakeholder groups, such as NGOs, veterinary and health professionals, and relevant businesses and private actors, in order to uncover a greater variety of perspectives and possibly gain more insight into the differences between the views of different stakeholder groups.

In addition, greater awareness of the capacity and willingness of potentially relevant actors to respond to climate change-induced infectious disease risk can facilitate cooperative and multi-stakeholder policy processes in this sector. A cooperative and integrated approach towards climate adaptation for health under the coordination of the European Union appears to be an important element of European governance of climate change and adaptation for health [12,13].

The call for responses to the health and also in particular infectious disease outcomes of climate change is becoming increasingly strong at a European level [12,13,14]. In response to this, this study focused on adaptation responses addressing climate change-induced infectious disease risk in Western Europe. A clear need for response strategies to climate change-induced infectious disease risk (and broader health outcomes) is emphasized at a European policy level. Yet, the remaining uncertainties on the effective use of adaptation (and mitigation) responses demonstrate that decision-making regarding this can prove to be a difficult task. The exploration of expert perspectives on adaptation responses using assessment criteria, as well as relevant actors’ willingness and capacity to respond can contribute to our understanding and the governance of the complex issue of climate change and its health outcomes. In order to address existing research gaps, a continued effort integrating multiple disciplines and research methods complementing participatory research approaches (such as modeling and scenario analysis) will be necessary.

## References

[1] IPCC (2013). Summary for Policymakers.

[2] Kovats R.S., Valentini R., Bouwers L.M., Georgopoulou E., Jacob D., Martin E., Rounsevell M., Soussana J.-F. (2014). Europe.

[3] Semenza J., Menne B. (2009). Climate change and infectious diseases in Europe. Lancet.

[4] WHO-Europe (2010). Climate change and health in Europe: Opportunities for action in partnership. Protecting Children’s Health in a Changing Environment; Proceedings of the Fifth Ministerial Conference on Environment and Health.

[5] Menne B., Apfel F., Kovats S., Racioppi F. (2008). Protecting Health in EUROPE from Climate Change.

[6] European Environment Agency (EEA) (2012). Climate Change, Impacts and Vulnerabilty in Europe 2012: An Indicator-Based Report.

[7] WHO-Europe Strategic Objective 1 to Reduce the Health, Social and Economic Burden of Communicable Diseases.

[8] Climate change in Europe. http://www.ecdc.europa.eu/en/healthtopics/climate_change/Pages/index.aspx.

[9] Martens P., Chang C.T., McEvoy D. (2012). Integrating adaptation and mitigation to climatic changes. Regions.

[10] Wardekker J.A., de Jong A., van Bree L., Turkenburg W.C., van der Sluijs J.P. (2012). Health risks of climate change: An assessment of uncertainties and its implications for adaptation policies. Environ. Health.

[11] WHO-Europe (2010). Parma Declaration on Environment and Health.

[12] European Commission (EC) (2013). Adaptation to Climate Change Impacts on Human, Animal and Plant Health.

[13] European Commission (EC) (2009). Adapting to Climate Change: Towards a European Framework for Action.

[14] European Commission (EC) Declaration of the European Commission. Proceedings of the Fifth Ministerial Conference on Environment and Health.

[15] European Commission (EC) (2007). White Paper: Together for Health: A Strategic Approach for the EU 2008–2013.

[16] Smith K.R., Woodward A., Campbell-Lendrum D., Chadee D.D., Honda Y., Liu Q., Olwoch J.W., Revich B., Sauerborn R. (2014). Human Health: Impacts, Adaptation, and Co-Benefits.

[17] (2005). MEA. Ecosystems and Human Well-Being: Policy Responses.

[18] McIntyre K.M., Setzkorn C., Baylis M., Waret-Szkuta A., Caminade C., Morse A.P., Akin S., Huynen M., Martens P., Morand S. (2010). Impact of climate change on human and animal health. Vet. Rec..

[19] De Ridder W., Turnpenny J., Nilsson M., von Raggamby A. (2007). A framework for tool selection and use in integrated assessment for sustainable development. J. Environ. Assess. Policy Manag..

[20] Kloprogge P., van der Sluijs J.P. (2006). The inclusion of stakeholder knowledge and perspectives in integrated assessment of climate change. Clim. Chang..

[21] Akin S., Martens P. (2014). A survey of Dutch expert opinion on climatic drivers of infectious disease risk in Western Europe. Climate.

[22] Prell C., Hubacek K., Reed M. (2013). Stakeholder analysis and social network analysis in natural resource management. Soc. Nat. Resour. Int. J..

[23] Reed M.S., Graves A., Norman D., Posthumus H., Hubacek K., Morris J., Prell C., Quinn C.H., Stringer L.C. (2009). Who’s in and why? A typology of stakeholder analysis methods for natural resource management. J. Environ. Assess. Policy Manag..

[24] Allen W., Kilvington M. (2003). Stakeholder Analysi.

[25] Davie T., Fenemore A., Allen W., Phillips C. (2006). Stakeholder Involvement in Integrated Catchment Management—Motueka, New Zealand.

[26] Freeman R.E., McVea J. (2001). A Stakeholder Approach to Strategic Management, Working Paper No. 01–02.

[27] Cooper D.R., Schindler P.S. (2003). Business Research Methods.

[28] Garson G.D. (2012). Sampling. Blue Book Series.

[29] WHO-Europe (2010). Protecting health in an environment challenged by climate change: European regional framework for action. Contribution of the Climate Change and Health Task Force.

[30] WHO-Europe (2010). Climate Change and Health: Policy Options for Climate Change and Health.

[31] IPCC (2007). Appendix I Glossary.

[32] ENHANCE Research Project. http://www.liv.ac.uk/enhance/.

[33] Füssel H.-M. (2007). Adaptation planning for climate change: Concepts, assessment approaches, and key lessons. Sustain. Sci..

[34] Ebi K., Kovats R.S., Menne B. (2006). An approach for assessing human health vulnerability and public health interventions to adapt to climate change. Environ. Health Perspect..

[35] WHO (2008). The Global Burden of Disease: 2004 Update.

[36] Nordhaus W.D. (1994). Expert opinion on climatic change. Am. Sci..

